# Survival analysis and clinical abnormalities in cats with progressive or regressive feline leukemia virus (FeLV) infection in Brazil

**DOI:** 10.1371/journal.pone.0322691

**Published:** 2025-07-01

**Authors:** Giovana Biezus, Thierry Grima de Cristo, Claudia Maria Flores Koehler, Flavia Yasmin de Quadros Kaveski, Thaís Sarria Viana Miranda, Paulo Eduardo Ferian, Renata Assis Casagrande

**Affiliations:** Departament of Veterinary Medicine, Universidade do Estado de Santa Catarina (UDESC), Av. Luís de Camões, Lages, Santa Catarina, Brazil; University of Bari, ITALY

## Abstract

This study aimed to characterize the clinical presentations and effects of progressive and regressive outcomes of feline leukemia virus (FeLV) infection on the life expectancy of cats. In total, 176 cats were selected: 116 with progressive infection (FeLV^+^P), 30 with regressive infection (FeLV^+^ R), and 30 FeLV-negative cats (Control). The cats underwent testing using ELISA to detect the FeLV p27 antigen and nested polymerase chain reaction to identify U3-LTR region and *gag* proviral DNA. The cats were clinically monitored until their death or for a period ranging 12–54 months. Survival analysis was performed using Kaplan–Meier analysis and Cox regression. The median survival time following FeLV diagnosis was 30 days for the FeLV^+^P group. The median survival time was not reached for the other groups. The cats’ health status (sick) at the time of inclusion in the study and the progression status of the FeLV infection led to a 4–5-fold increase in the Hazard Ratio (HR) for death in the general population. The primary causes of death among cats in the FeLV^+^P group were lymphoma, leukemia, anemia, and other diseases. In the FeLV^+^R group, the causes of death included leukemia, anemia, and other diseases. Progressive FeLV infection reduced life expectancy, whereas regressive FeLV infection had no direct impact on the survival curve.

## Introduction

Feline leukemia virus (FeLV), considered one of the most harmful viruses to cat health, is prevalent in various cat populations worldwide [1–4]. Some countries have managed to contain the spread of this virus, while in Brazil, the prevalence rates remain high, making the Brazilian cat population ideal for investigating the clinical features of the disease [3,5–8]. Previous studies estimated disease prevalence in Brazil ranged from 28.41% to 45.6% [3,8]. In Germany and North America, a prevalence of 3–3.6% and 3.1% were found, respectively [5–7]. The different prevalence values are due to program detection implementation, cat removal and vaccination decreased the prevalence of FeLV [9].

FeLV can cause progressive, regressive, and abortive infections in the host [10]. All these outcomes can be observed in natural FeLV infections and are influenced by the host’s immune capacity to control viremia [4,11–13].

Progressive infection arises when the host immune response is ineffective against the virus, leading to the insertion of the provirus into the cat genome and the development of persistent viremia. Regressive infection arises when the host immune response is partially effective, fighting FeLV at the onset of its spread to the bone marrow, thus preventing persistent viremia but not the integration of proviral DNA. In abortive infection, the virus is neutralized before it reaches the bone marrow. Cats with abortive infections are considered to have been exposed to the virus, and the sole indication of this is the production of antibodies against FeLV [10,14].

Only progressive infections demonstrated clinical severity [8]. Regressive infection has a suggested clinical effect, but few studies confirm that FeLV-regressive infection influences the health status of cats [14–16].

Cats with progressive infection have a low life expectancy, surviving approximately 3 years after being infected with the virus [17]. This is due to the development of clinical conditions such as lymphoma and leukemia; non-neoplastic hematological disorders; and immunodeficiency, which increases susceptibility to concomitant infections [17–23]. Among the main concomitant infections affecting these cats is the feline immunodeficiency virus (FIV), which is also the main differential diagnosis [10].

In regressive infections, the primary concern for cat health is the provirus’s ability to reactivate during episodes of immunodeficiency, leading to progressive infections. Another concern is the ability of proviral DNA to interact with cellular proto-oncogenes through somatically acquired insertional mutagenesis. This can lead to neoplasms even in older cats [14,24]. Additionally, cats with regressive infection can transmit FeLV through blood transfusions, and the recipient cat may become viremic [16]. It is possible that up to 25% of cats exposed to FeLV in intense infectious pressure areas may develop regressive infections. [10].

There is a lack of Brazilian and other countries studies that consider the clinical monitoring of cats with FeLV infection, particularly those that address the two main outcomes of the infection. Therefore, the aim of this study was to characterize the clinical presentations and the effects of progressive and regressive FeLV infection on the life expectancy of cats.

## Materials and methods

### Sample selection

Cats were selected from routine care at the Veterinary Hospital of the Santa Catarina State University (UDESC) between November 2015 and August 2019. In total 384 cats were part of two previous cross-sectional studies on progressive and regressive FeLV infections in the Santa Catarina Plateau, Brazil [3–8]. Healthy cats (from routine examinations or surgeries for elective neutering) and sick cats were included in their studies, without distinction of age, sex, or breed. For this study, another inclusion criterion was that the cat should be available for monitoring for at least 12 months before the study was due to end. The cats were tested for the presence of FIV antibodies and FeLV antigens using ELISA, using the SNAP FIV/FeLV Combo Test® kit (IDEXX Laboratories, Maine, USA). Nested-PCR was also performed according to a technique adapted [8] using sense primers in the U3 region of LTR, and antisense primers in the gag region [25]. Both tests were performed with blood samples collected on the same day. The project was approved by the Ethics Committee for Animal Use (CEUA 5806100918).

### Determining the outcome of FeLV infection

Cats that tested positive for FeLV by ELISA and nested-PCR were considered to have progressive infection; cats that tested positive for FeLV by nested-PCR but not ELISA were considered to have regressive infection [8,10]. Cats that were not diagnosed with the infection by ELISA and nested-PCR were considered negative for FeLV.

Based on these criteria, the cats were divided into three groups: 116 FeLV-infected cats with progressive infection (FeLV^+^ P); 30 FeLV-infected cats with regressive infection (FeLV^+^ R); 30 FeLV-negative cats (control). No distinction was made based on breed, age, sex, health status, or co-infection with FIV. ELISA and nested-PCR tests were again carried out in the FeLV^+^ P and FeLV^+^ R cats that were alive at the end of the study to detect any changes in the infection outcome.

### Clinical monitoring

The cats in the three groups were clinically monitored, either in person or by telephone, every six months from the time they were included in the study until their death or the end of the study period.

Veterinary workers conducted face-to-face monitoring based on each cat’s individual needs and their owner’s availability. The cats underwent physical examinations, blood counts, and complementary imaging and laboratory tests when necessary. In the event of death during the monitoring period, the cats underwent necropsy and histopathological examination with their owners’ authorization.

The animals’ deaths occurred from debilitating terminal illnesses, which can or are not associated with FeLV. In some cases, the veterinarian may recommend euthanasia to relieve the animal’s suffering. Euthanasia was just realized in cases of debilitating terminal illnesses with owner consent.

For euthanasia, methadone (0,2 mg/kg) and midazolam (0,3 mg/kg, IM) intramuscular (IM) administration were used for pre-medication, propofol (2–8 mg/k) intravenous (IV) administration for induction and, potassium chloride (dose-effects) IV for cardiac arrest.

### Survival analysis

For the survival analysis, we initially employed a non-parametric approach using Kaplan–Meier analysis [26]. The observation period defined for the survival analysis was 2 years (730 days). Time was defined as the number of days elapsed from time zero (x-axis) to the occurrence of the event. The event was treated as a binary variable (0, 1), where 1 indicates death and 0 represents censored data (study abandonment, loss of information, or non-occurrence of death after 2 years of monitoring). Cats that died by euthanasia were classified in variable 1 once the euthanasia was just realized in cases of debilitating terminal illnesses. Survival curves were compared using the Log-Rank, Gehan–Breslow–Wilcoxon, and Tarone–Ware tests, and *p* < 0.05 was considered to indicate a statistically significant difference [27].

Given the proportionality of the risks, the Cox regression model was applied and *p* < 0.05 was considered to be statistically significant [28,29]. The variables used for the analysis included: age at the time of inclusion in the study, health status at the time of inclusion health status at the time of inclusion, outcomes of FeLV infection (FeLV^+^P, FeLV^+^R, and control groups), and co-infection with FIV. Both analyses were performed using SPSS^®^ (version 25, IBM^®^, NY, USA).

### Analysis of clinical data

The medical records of the cats in the FeLV^+^ P and FeLV^+^ R groups were evaluated. The data collected from consultations and additional tests, from the time of inclusion in the study until the time of death or the end of the study, were organized into contingency tables. Data such as age, breed, sex, and clinical signs exhibited by the cats were collected.

The health status observed at the time of inclusion in the study and at the time of death were compared and cross-checked with the results of histopathological examinations, when available.

## Results

### Sample

Of all the cats that participated in the study (176), 92% and 8% were classified as mixed breed (MB) and pure breed, respectively. Regarding gender, 58% were male and 42% were female. The data collected for the subsequent survival analysis and clinical monitoring are presented in Table 1.

**Table 1:** The frequency of the total cats across the groups (FeLV^+^P, FeLV^+^R, and control) based on the variables analyzed in the survival analysis and the clinical monitoring.

Variables		FeLV^ + ^P n = 116	FeLV^ + ^R n = 30	Control n = 30
**Age of cats at the time of inclusion in the study**	median (25–75%) in days	1003 (547-1825)	698 (365-1825)	1460 (730-3011)
**FIV-coinfected**	Yes (%)	8 (6.9)	4 (13.3)	0
	No (%)	108 (93.1)	26 (86.7)	30 (100)
**Health status at the time of inclusion in the study**	Sick (%)	100 (86.2)	11 (36.7)	17 (56.7)
	Healthy (%)	16 (13.8)	19 (63.3)	13 (43.3)
**Life status at the end of the study**	Death (%)	103 (88.8)	9 (30)	9 (30)
	Alive (%)	10 (8.6)	14 (46.7)	15 (50)
	Unknown (%)	3 (2.6)	7 (23.3)	6 (20)
**Euthanasia *in extremis***	Yes (%)	24 (23.3)	1 (11.1)	2 (22.2)
	No (%)	79 (76.7)	8 (88.9)	7 (77.8)
**Death due to suspected FeLV-associated disease**	Yes (%)	70 (68.0)	4 (44.5)	1 (11)
	No (%)	4 (3.9)	1 (11)	4 (44.5)
	Undetermined (%)	29 (28.1)	4 (44.5)	4 (44.5)

FeLV^+^P: progressive FeLV infection; FeLV^+^R: regressive FeLV infection.

In the FeLV^+^P group, 2.59% (3/116) of the cats had an unknown life status at the end of the study. Of these, two ran away from home after 30 and 487 days of monitoring, respectively, and one lost contact with its owner after 730 days. In the FeLV^+^R group [23.33% (7/30)], five cats ran away from home between 30 and 1217 days of monitoring, while two lost contact with their owners after 760 and 821 days, respectively. In the control group, [20.0% (6/30)] lost contact with their owners after 152–791 days of monitoring.

### Survival analysis

Of all the cats that died during the study, 3.9% (4/103) from the FeLV^+^P group and 30.0% (3/9) from the control group died after 730 days. These cats were considered to be alive in the survival analysis. Of all the cats alive at the end of the study, 60.0% (6/10) in the FeLV^+^P group were monitored for less than 730 days, 50.0% (7/14) in the FeLV^+^R, and 60.0% (9/15) in the control group. These cats were excluded from the survival analysis.

All the cats in the FeLV^+^P group co-infected with FIV died before 730 days of monitoring, while only one in the FeLV^+^R group died.

**Fig 1:**
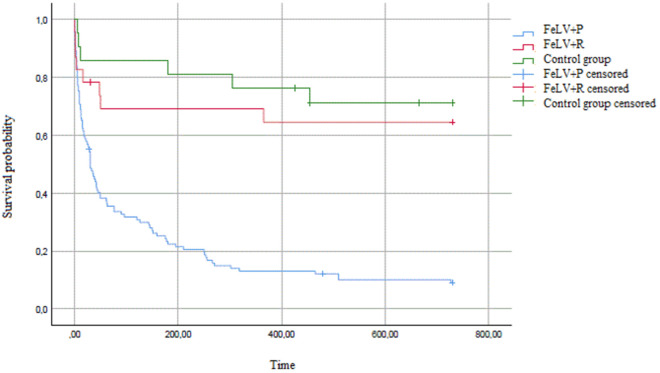
Kaplan–Meier survival curves obtained for cats with progressive FeLV infection (FeLV ^+^P; n = 110), cats with regressive infection (FeLV^+^R; n = 23), and the control group (n = 21). While the median survival time was not reached for the FeLV^+^R and control groups, the median survival time was 30 days for the FeLV^+^P group. The difference between the curves was statistically significant (*p* < 0.001).

The mean and median survival times of the groups (FeLV + P, FeLV + R, and control) are reported in Table 2 and in Fig 1.

**Table 2:** Descriptive statistics of number of deaths, number of censored dates, and estimated mean and median survival time (days) obtained for cats with progressive FeLV infection (FeLV^+^P: n = 110), cats with regressive infection (FeLV^+^R: n = 23), and the control group (n = 21) calculated using Kaplan Meier analysis.

	Group		
	FeLV^ + ^P	FeLV^ + ^R	Control
**n. of events (death)**	99/110	8/23	6/21
**n. of censored**	11/110	15/23	15/21
**n. of alive cats**	8	8	9
**n. of cats lost to follow up**	3	7	6
**Estimated mean survival time (95.0% CI)**	141.5 (99.14-183.85)	492.71 (357.50-627.92)	566.51 (449.56-683.45)
**Estimated median survival time (95.0% CI)**	30.0 (20.8-39.16)	–	–

The median survival time following diagnosis was 30 days (20.80–39.16 days) for the FeLV^+^P group, while the median survival time was not reached for the FeLV^+^R and control groups. The status of progressive FeLV significantly affected cat longevity, with *p* < 0.001 in the Log-Rank, Gehan–Breslow–Wilcoxon, and Tarone–Ware tests. The comparison of the Kaplan–Meier survival curves for the three groups is shown in Fig 1.

Cox regression revealed that the cats’ health status at the time of inclusion in the study and the outcome of the FeLV infection significantly influenced the hazard ratio (HR) for death in cats over time (p < 0.001). Cats that were already sick at the time of inclusion were 4 times more likely to die and 5.5 times in the case of FeLV^+^P. The HR and *p*-values estimated by Cox regression are presented in Table 3.

**Table 3:** Hazard ratios (HR) and *p*-values estimated by Cox regression for variables associated with the survival curves of the cats.

Variables		COX regression		
		n	*p* value	HR (95% CI)
**Age**			0.59	1.00 (0.99-1.01)
**Sick cats**	Yes	124	**<0.001**	**4.05 (1.95-8.39)**
	No	30	–	–
**FIV-coinfected**	Yes	12	0.90	0.95 (0.49-1.86)
	No	142	–	–
**Groups**	FeLV^ + ^P	110	**<0.001**	**5.47 (2.31-12.91)**
	FeLV^ + ^R	23	0.16	2.18 (0.74-5.69)
	Control	21	–	–

CI: confidence interval

The same variables underwent Cox regression for each study group. The health status significantly increased the average HR for the outcome (death) over time in the FeLV^+^P (*p* = 0.05) and FeLV^+^R (*p* = 0.02) groups. Cats in the FeLV^+^P and FeLV^+^R groups, which were already sick at the time of inclusion in the study, were 3 and 13 times more likely to die than healthy cats, respectively. The HR and *p*-values estimated by Cox regression are presented in Table 4.

**Table 4:** Hazard ratios (HR) and *p*-values estimated by Cox regression analysis for covariates associated with the survival curves of the cats belonging to the FeLV^+^P (n = 110), FeLV^+^R (n = 23), and control (n = 21) groups.

Variables		COX regression					
		FeLV^ + ^P		FeLV^ + ^R		Control	
		*p* value	HR (95% CI)	*p* value	HR (95% CI)	*p* value	HR (95% CI)
**Age**		0.50	1.0 (0.99-1.01)	0.36	1.0 (0.99-1.02)	0.39	1.01 (0.99-1.03)
**Sick cats**	Yes	0.05	3.09 (1.41-6.75)	0.02	13.33 (1.43-124.03)	0.39	2.75 (0.27-28.48)
	No	–	–	–	–	–	–
**FIV-coinfected**	Yes	0.55	1.25 (0.6-2.60)	0.29	0.32 (0.04-2.68)	–	–
	No	–	–	–	–	–	–

CI: confidence interval

### Clinical monitoring

Of the cats that died in the FeLV^+^P group and for whom the cause of death could be determined (n = 74), the disease present at the time of the cats’ inclusion in the study was deemed responsible for the deaths in 89.2% of cases, while for 10.8% of cases, the causes were different. Among the diseases, 79.73% (59/74) are strongly associated with FeLV. For 20.27% (15/74), there is poor or no association with FeLV. These diseases are feline gingivitis-stomatitis complex (FCGS), fecaloma, secondary infections, polytrauma, and some neoplasms such as malignant nerve sheath tumor, histiocytoma and laryngeal fibrosarcoma. In the FeLV^+^R (n = 5) and control (n = 5) groups, the clinical abnormalities observed at the time of inclusion were responsible for the deaths in all cases. Of the cats that died in the FeLV^+^R group, two diseases have no association with FeLV, the squamous cell carcinoma and viral coinfection. In the control group, there are no deaths associated with FeLV. The data on the diagnosis of the observed clinical manifestations at death are presented in Table 5.

**Table 5:** Frequency of clinical manifestations in cats with progressive (FeLV^+^P) and regressive (FeLV^+^R) FeLV infections and control groups at the moment of the death.

Clinical manifestations	FeLV^ + ^P (n = 74)	FeLV^ + ^R (n = 5)	Control (n = 5)
**Anemia**	12	1	–
**Bone marrow aplasia**	1	–	–
**FCGS**	9^*^	–	–
**FLUTD**	–	–	1
**Fecaloma**	1	–	–
**FIV**	3^*^	2^*^	–
**Liver Disease**	–	–	1
**Bacterial infections**	5 + 1^*^	1^*^	–
**Fungal infections** ^1^	–	–	1
**Parasitic infections** ^2^	1^*^	–	–
**Lymphoma**	29	–	–
**Leukemia**	18	2	–
**Other neoplasms** ^3^	3 + 1^*^	1	1
**FIP**	5	1	1
**Polytrauma**	1	–	–
**Total**	74 + 15^*^	5 + 3^*^	5

FCGS: feline gingivitis-stomatitis complex; FLUTD: feline lower urinary tract disease; FIV: feline immunodeficiency virus; FIP: feline infectious peritonitis;

* concomitant clinical abnormalities.

^1^ cryptococcosis;

^2^ pneumonia caused by *Aelurostrongylus* sp;

^3^ FeLV + P: malignant nerve sheath tumor, histiocytoma, laryngeal fibroma, laryngeal fibrosarcoma;

^3^ FeLV^+^R: squamous cell carcinoma;

^3^ Control: squamous cell carcinoma.

Among the diseases mentioned above and diagnosed in more than one group, just the ones strongly associated with FeLV are described here. Lymphoma was the most reported neoplastic disease and the clinical monitoring data are presented in Table 6.

**Table 6:** The frequency of cats diagnosed with lymphoma in FeLV^+^P group based on the population data end the clinical manifestations.

Population data		% (n = 29)
Age of cats at the time of death	median (25- 75%) in days	1095 (547 −1923)
**FIV-coinfected**	Yes	93.1 (27)
	No	6.9 (2)
**Type of lymphoma**	Alimentary	3.4 (1)
	Extranodal^*^	13.8 (4)
	Mediastinal	62.1 (18)
	Multicentric	20.7 (6)

FeLV + P: progressive FeLV infection;

* the extranodal lymphomas included one in the nasal cavity, another in the spinal cord between L2 and L3, and two subcutaneous lymphomas.

The diagnosis of lymphoma was confirmed in 10.34% (3/29) of cases by pleural effusion analysis, in 24.14% (7/29) of cases by fine needle aspiration (FNA), and in 20.69% (6/29) of cases by histopathological examination. For the remaining cats [44.83% (13/29)], the diagnosis was made using two or more methods in combination.

For leukemia cases in the FeLV^+^P group, the median age at death was 1460 days (range: 730–1916 days). Histopathological analysis was performed on 55.6% (10/18) of the leukemia cases in the FeLV + P group, and they were classified into acute myeloid leukemia [40.0% (4/10)], chronic myeloid leukemia [30.0% (3/10)], and acute lymphoid leukemia [30.0% (3/10)]. For the remaining cases [44.4% (8/18)], diagnosis was made through blood tests and by ruling out any signs of lymphoma during physical examination.

The cats with leukemia (n = 2) in the FeLV^+^R group died at 2555 and 5840 days old. They underwent histopathological evaluation and were diagnosed with chronic myeloid leukemia and acute lymphoid leukemia.

Among anemia cases in the FeLV^+^P group, the median age at death was 547 days (range: 365–1916 days). One of the cases was co-infected with FIV. Non-regenerative anemia [packed cell volume – median: 7%, and range: 6–11% (reference range: 24–45%); reticulocyte count – median: 0/μL, and range: 0–9.2 x10^3^/μL (reference range: ≥ 65.00)] were observed in 91,67% (11/12) cases, just one case was regenerative (packed cell volume: 9%; reticulocyte count: 160.8x10^3^/μL) [30]. Anemia was not the only hematological abnormality observed in these cats. Cytopenia were observed in two or more cell lines in 91.67% (11/12) of the cases, platelet line being the most affected (75%), followed by the white blood cells (63,6%). A single erythrocyte abnormality was observed in only one case.

In the FeLV + R group, the anemic cat died at 657 days old with cytopenia in two cell lines: red blood cells (packed cell volume: 8%; reticulocyte count: 0/µL), accompanied by neutropenia (segmented neutrophils count: 751/µL).

In both groups, a complete blood count was available to diagnose anemia and rule out any signs of lymphoma, leukemia or other diseases.

The median age at death in Control group were 4015 days (range: 2810–4380 days).

### Controlling the outcome of the infection

In the FeLV^+^P and FeLV^+^R groups, 10.3% (12/116) and 46.7% (14/30) of the cats, respectively, underwent retesting with ELISA and nested-PCR for periods ranging from 426 to 1703 months. All the cats in the FeLV^+^P group continued to test positive using both diagnostic methods. Among FeLV^+^P cats, 75% (9/12) were alive at the end of the study, while 25% (3/12) had died. In the FeLV^+^R group, 42.9% (6/14) of the cats remained positive only by nested-PCR. Of these, 50% (3/6) remained healthy at the end of the monitoring period, while the others were diagnosed with FIV, lymphoma, and FGSC. In the FeLV^+^R group, 57.1% (8/14) of the cats tested negative in both tests. This group included two FIV-positive cats, while the remaining cats were healthy. In the FeLV^+^R group, no one cat died at the end of the study period. The cats that were not retested had died before the end of the study or were not clinically monitored in person.

## Discussion

This study enabled the determination of the impact of progressive (FeLV^+^P) and regressive (FeLV^+^R) outcomes of natural FeLV infection in a cat population. Few studies have adopted this approach, with most assessing survival time solely for the progressive outcome [7,19,31–35].

The high mortality rate observed in this study over a short period is attributed to the fact that the sample was drawn from a hospital population, where most of the cats already had some clinical abnormality. This is reflected in the regression analysis, where health status (being sick) and progressive FeLV infection were found to increase the HR for death. It should be emphasized that in this study, there was no separation between sick and healthy cats within the groups, and many diseases can be cured with proper treatment. However, when progressive FeLV infection comes into play, life expectancy is invariably reduced, and few cats survive for long periods due to the development of fatal diseases [31,34,36].

The median survival time in the FeLV^+^P group was shorter than that observed in other countries, where FeLV screening is routinely performed on healthy cats [5–7]. The cats in this study often underwent the diagnostic test after the clinical syndrome related to FeLV had already appeared [5,34,35].

The low median survival time in the FeLV^+^P group also depends on the host population characteristics: high host density, increased access to the outdoors, cohabitation with other animals, and low vaccination coverage for FeLV all contribute to the virus’s persistence and spread within the population [8]. This result is also supported by veterinary care data from a previous study conducted on the same population [3]. In that study, most owners reported taking their cats to the veterinarian only when they became ill (32.85%), while 49.27% had visited the veterinarian once during the study period [3].

Cats naturally infected with regressive infection (FeLV^+^R) have a life expectancy like uninfected cats, as also observed in another study [35]. The same results were observed in experimental infections, where cats with regressive infection lived longer than those with progressive infection. In some instances, these cats were monitored for up to 12 years without developing any FeLV-associated diseases [19].

Although the regressive infection did not affect the life expectancy of the cats in this study, we cannot rule out the possibility that it might directly impact the health of these cats. In the individual assessment of the groups, the health status influenced the HR of the cats with both progressive and regressive infections. In the FeLV^+^R group, *p*-value was more significant than the FeLV^+^P group. In the FeLV^+^P group, most of the cats were already sick at the time of inclusion in the study and therefore died until the end. In the FeLV^+^R group, most of the cats were healthy. Even so, 5 cats died at the end of the study. These facts may have influenced the difference between *p*-values and HR results. Supporting these findings, studies have indicated that cats with regressive infection can develop fatal diseases commonly associated with FeLV, including non-regenerative cytopenias and lymphoma [15,37,38]. These diseases are reported to be associated with regressive outcomes, occurring at varying rates [13,32,33,39].

FIV co-infection did not significantly influence the HR for death in the cats in this study, an unexpected finding for both groups of cats. It is likely that the fact most of the cats were already sick at the time they were included in this study influenced this result. For cats with regressive FeLV infection, it is important to note that FIV infection follows a chronic course, and the regressive outcome of FeLV appears to be more detrimental to the cats’ long-term health [37–40]. However, this was not observed in our study due to the short monitoring period.

Although younger cats are more susceptible to progressive FeLV infection [41], no correlation was found between the age at FeLV diagnosis and survival time, since most cats, regardless of their age, already had some FeLV-related disease. In an experimental study, cats had an average lifespan of 3.1 years before falling sick and being euthanized [19]. In the aforementioned study, the exact moment of infection is established [19]. However, in cases of natural infection, as observed in the present study, it may have occurred months or even years prior to the diagnosis. This makes analyzing cause and effect over time difficult.

In cases where the clinical cause of death was identified, it is possible to observe a difference in the distribution of age at death among cats within each group. While the FeLV^+^P cats died at ages ranging from 212 days to 2190 days (6 years), the FeLV-negative cats were older (2555–4380 years). Another study obtained similar results, showing that 80% of FeLV-positive cats died by the age of 1277 days (3.5 years), while only 10% of FeLV-negative cats did so [5]. In the FeLV^+^R group, deaths occurred across a wide age range, from young cats to adults and seniors, which is different from findings reported in the literature, where diseases primarily develop in cats older than 2190 days (6 years) [32,37–39].

Some of the diseases observed in this study are strongly associated with FeLV, especially lymphoma and leukemia. Mediastinal and multicentric lymphomas are more common in cats with FeLV, but alimentary and extranodal lymphomas have also been reported [22,23,42]. The anatomical locations of the extranodal lymphomas are noteworthy, especially because there were two cases of subcutaneous lymphoma. Nasal and central nervous system lymphomas are common types of extranodal lymphomas (46.3% and 10.1% of cases, respectively), and are associated with FeLV in up to 56.5% of instances. In contrast, subcutaneous lymphoma is rarely seen in cats (3.4 to 4.5% of cases), with its association with FeLV varying from 6.5% to 58.8% [42–47].

The association of leukemia, the second most common neoplastic disease, with progressive FeLV infection is well-established, with up to 87.5% of cases of FeLV infection also showing leukemia [21]. Both myeloid and lymphoid leukemia can be diagnosed in cats, especially in those that are juveniles and young adults [21,23], as observed in the present study.

In general, leukemia occurred at a low rate in cats with regressive infection. In these cases, the oncogenic potential of FeLV is linked to the persistence of proviral DNA sequences integrated into the host genome, coupled with a decline in immune surveillance of cells as the cats age [38]. This fact, along with the young age of the cats in the FeLV^+^R group and the short monitoring period of this study, accounts for the result.

Similar to this study, other studies have demonstrated that anemia is one of the main causes of death in cats with progressive FeLV infection [17,34,48]. Non-neoplastic mechanisms involved in the development of FeLV-associated anemia include the primary infection of hematopoietic stem cells and stromal cells, which leads to non-regenerative anemia [14,49,50].

Non-regenerative anemia associated with FeLV is typically observed in cases of bone marrow aplasia, pure red cell aplasia, and myelodysplastic syndrome (MDS) [32]. Bone marrow aplasia is associated with the FeLV-C subgroup, as is pure red cell aplasia [51,52]. The first is characterized by a deficiency in the production of erythroid, myeloid, and platelet lines in the bone marrow [50,53]. While, pure red cell aplasia leads to non-regenerative anemia without other cytopenias [45], as observed in the present study. MDS is characterized by a combination of cytopenias in the peripheral blood, usually affects two or more cell lines, and is a condition that precedes acute myeloid leukemia [54]. Most of the cats with anemina reported here had cytopenias in two or more cell lines which is a condition that precedes acute myeloid leukemia [54]. However, a definitive diagnosis of pure red cell aplasia and MDS can only be made by histopathological evaluation [53,55,56]. Additionally, the occurrence of anemia, whether regenerative or not, can be attributed to immune-mediated hemolytic anemia caused by FeLV. This condition arises from the expression of foreign antigens on the surface of red blood cells, resulting in their destruction [57].

Cats that initially test positive for both the p27 antigen and the FeLV provirus can transition to a regressive infection, usually within 16 weeks of infection [10]. In this study, no cat with progressive infection showed a regressive outcome over time. Another study evaluating natural FeLV infection showed similar results [13]. The hypothesis is that FeLV infection was detected in these cats at a later stage, by which time the host had already developed an ineffective immune response, resulting in progressive infection [58]. The main limitation of this study was the small number of cats retested. However, these cats died of FeLV-disease-associated before the end of the study period and probably did not transition to a regressive infection.

In many cats with regressive infection, it was no longer possible to detect proviral DNA. This was primarily observed in cats that underwent follow-up examinations after longer periods. This was not observed in an experimental study, where cats with regressive FeLV infection were kept in a controlled environment without exposure to viremic cats for more than 6 years, yet FeLV was still detectable in most of their tissues [19]. There are three possible explanations for the observed results. The first hypothesis is that over time, some cats are able to either eliminate or suppress the population of infected cells, eventually resembling cats with abortive infection [59]. The second hypothesis is that if these cats are exposed to new samples at various times, they may eventually show positive results [58,60]. Finally, it cannot be ruled out that the nested-PCR was not sensitive enough to detect extremely low levels of proviral DNA. A similar issue has been reported previously, in which multiple PCR assays were required on the same sample to yield a positive result. This suggests that the proviral loads were at the limit of the assay’s detection capability [58]. In this study, the samples were retested and identical results were obtained.

Even though it has not been observed, the possibility of infection reactivation in cats with regressive outcome should still be considered [58]. This risk would be higher in cats monitored for a short period, as the risk of reactivation tends to decrease over the years following infection [19].

## Conclusions

This study demonstrated the devastating impact of progressive FeLV infection and provided evidence of the actual impact of regressive infection in populations with high prevalence of FeLV, where control and prevention measures are not effectively implemented.

Survival analysis shows that progressive FeLV infection reduces the life expectancy of cats, while regressive FeLV infection does not directly affect their survival curve. The clinical evaluation showed that while clinical abnormalities such as lymphoma, leukemia, and non-regenerative anemia are more common in cats with progressive infection, they also frequently occur in those with regressive infection.

## Supporting information

S1 File [Survival analysis_FeLVP]. [Pages 1–4: Cox regression analysis for covariates associated with the survival curves of the cats belonging to the FeLV + P (n = 110) group]. [Variables in the equation: Age of cats at the time of inclusion in the study; Health status cats at the time of inclusion in the study; FIV co-infection].
(PDF)

S2 File [Survival analysis_FeLVR and Control]. [Pages 1–3: Cox regression analysis for covariates associated with the survival curves of the cats belonging to the FeLV + R (n = 23)], [Pages 4–8: Cox regression analysis for covariates associated with the survival curves of the cats belonging to the control (n = 21) group]. [Variables in the equation: Age of cats at the time of inclusion in the study; Health status cats at the time of inclusion in the study; FIV co-infection].
(PDF)

S3 File [Survival analysis_Total Cats]. [Pages 1–12: Kaplan–Meier analysis and survival curves obtained for cats with progressive FeLV infection (FeLV + P; n = 110), cats with regressive infection (FeLV + R; n = 23), and the control group (n = 21)]. [Pages 13–17: Cox regression analysis for covariates associated with the survival curves of the total cats]. [Variables in the equation: Age of cats at the time of inclusion in the study; Health status cats at the time of inclusion in the study; FIV co-infection; Groups in the study (FeLV + P, FeLV + R and Control)].
(PDF)

## References

[1] Galdo Novo S, Bucafusco D, Diaz LM, Bratanich AC. Viral diagnostic criteria for Feline immunodeficiency virus and Feline leukemia virus infections in domestic cats from Buenos Aires, Argentina. Rev Argent Microbiol. 2016;48(4):293–7. doi: 10.1016/j.ram.2016.07.003 27825735

[2] Sivagurunathan A, Atwa AM, Lobetti R. Prevalence of feline immunodeficiency virus and feline leukaemia virus infection in Malaysia: a retrospective study. JFMS Open Rep. 2018;4(1):2055116917752587. doi: 10.1177/2055116917752587 29568541 PMC5858631

[3] Biezus G, Machado G, Ferian PE, da Costa UM, Pereira LHH da S, Withoeft JA, et al. Prevalence of and factors associated with feline leukemia virus (FeLV) and feline immunodeficiency virus (FIV) in cats of the state of Santa Catarina, Brazil. Comp Immunol Microbiol Infect Dis. 2019;63:17–21. doi: 10.1016/j.cimid.2018.12.004 30961813

[4] Giselbrecht J, Jähne S, Bergmann M, Meli ML, Pineroli B, Boenzli E, et al. Prevalence of different courses of feline leukaemia virus infection in four European countries. Viruses. 2023;15(8):1718. doi: 10.3390/v15081718 37632060 PMC10459464

[5] Gleich SE, Krieger S, Hartmann K. Prevalence of feline immunodeficiency virus and feline leukaemia virus among client-owned cats and risk factors for infection in Germany. J Feline Med Surg. 2009;11(12):985–92. doi: 10.1016/j.jfms.2009.05.019 19616984 PMC11318771

[6] Burling AN, Levy JK, Scott HM, Crandall MM, Tucker SJ, Wood EG, et al. Seroprevalences of feline leukemia virus and feline immunodeficiency virus infection in cats in the United States and Canada and risk factors for seropositivity. J Am Vet Med Assoc. 2017;251(2):187–94. doi: 10.2460/javma.251.2.187 28671491

[7] Englert T, Lutz H, Sauter-Louis C, Hartmann K. Survey of the feline leukemia virus infection status of cats in Southern Germany. J Feline Med Surg. 2012;14(6):392–8. doi: 10.1177/1098612X12440531 22403413 PMC10822583

[8] Biezus G, Grima de Cristo T, da Silva Casa M, Lovatel M, Vavassori M, Brüggemann de Souza Teixeira M, et al. Progressive and regressive infection with feline leukemia virus (FeLV) in cats in southern Brazil: Prevalence, risk factors associated, clinical and hematologic alterations. Prev Vet Med. 2023;216:105945. doi: 10.1016/j.prevetmed.2023.105945 37209619

[9] Studer N, Lutz H, Saegerman C, Gönczi E, Meli ML, Boo G, et al. Pan-European Study on the prevalence of the feline leukaemia virus infection - reported by the European Advisory Board on Cat Diseases (ABCD Europe). Viruses. 2019;11(11):993. doi: 10.3390/v11110993 31671816 PMC6893802

[10] Little S, Levy J, Hartmann K, Hofmann-Lehmann R, Hosie M, Olah G, et al. 2020 AAFP feline retrovirus testing and management guidelines. J Feline Med Surg. 2020;22(1):5–30. doi: 10.1177/1098612X19895940 31916872 PMC11135720

[11] Hoover EA, Mullins JI. Feline leukemia virus infection and diseases. J Am Vet Med Assoc. 1991;199(10):1287–97. doi: 10.2460/javma.1991.199.10.1287 1666070

[12] Hofmann-Lehmann R, Huder JB, Gruber S, Boretti F, Sigrist B, Lutz H. Feline leukaemia provirus load during the course of experimental infection and in naturally infected cats. J Gen Virol. 2001;82(Pt 7):1589–96. doi: 10.1099/0022-1317-82-7-1589 11413369

[13] Westman M, Norris J, Malik R, Hofmann-Lehmann R, Harvey A, McLuckie A, et al. The Diagnosis of Feline Leukaemia Virus (FeLV) infection in owned and group-housed rescue cats in Australia. Viruses. 2019;11(6):503. doi: 10.3390/v11060503 31159230 PMC6630418

[14] Hartmann K. Clinical aspects of feline retroviruses: a review. Viruses. 2012;4(11):2684–710. doi: 10.3390/v4112684 23202500 PMC3509668

[15] Suntz M, Failing K, Hecht W, Schwartz D, Reinacher M. High prevalence of non-productive FeLV infection in necropsied cats and significant association with pathological findings. Vet Immunol Immunopathol. 2010;136(1–2):71–80. doi: 10.1016/j.vetimm.2010.02.014 20398945 PMC7112630

[16] Nesina S, Katrin Helfer-Hungerbuehler A, Riond B, Boretti FS, Willi B, Meli ML, et al. Retroviral DNA--the silent winner: blood transfusion containing latent feline leukemia provirus causes infection and disease in naïve recipient cats. Retrovirology. 2015;12:105. doi: 10.1186/s12977-015-0231-z 26689419 PMC4687292

[17] Lutz H, Addie D, Belák S, Boucraut-Baralon C, Egberink H, Frymus T, et al. Feline leukaemia. ABCD guidelines on prevention and management. J Feline Med Surg. 2009;11(7):565–74. doi: 10.1016/j.jfms.2009.05.005 19481036 PMC7172531

[18] Reinacher M. Diseases associated with spontaneous feline leukemia virus (FeLV) infection in cats. Vet Immunol Immunopathol. 1989;21(1):85–95. doi: 10.1016/0165-2427(89)90132-3 2549696 PMC7133624

[19] Helfer-Hungerbuehler AK, Widmer S, Kessler Y, Riond B, Boretti FS, Grest P, et al. Long-term follow up of feline leukemia virus infection and characterization of viral RNA loads using molecular methods in tissues of cats with different infection outcomes. Virus Res. 2015;197:137–50. doi: 10.1016/j.virusres.2014.12.025 25553598

[20] Biezus G, Ferian PE, Da Silva Pereira LHH, Withoeft JA, Antunes MM, Nunes Xavier MG, et al. Clinical and haematological disorders in cats with natural and progressive infection by Feline Leukemia Virus (FeLV). Acta Scientiae Vet. 2019;47(1). doi: 10.22456/1679-9216.90027

[21] Cristo TG, Biezus G, Noronha LF, Gaspar T, Dal Pont TP, Withoeft JA, et al. Feline leukaemia virus associated with leukaemia in cats in Santa Catarina, Brazil. J Comp Pathol. 2019;170:10–21. doi: 10.1016/j.jcpa.2019.05.002 31375152

[22] Cristo TG, Biezus G, Noronha LF, Pereira LHHS, Withoeft JA, Furlan LV, et al. Feline lymphoma and a high correlation with feline leukaemia virus infection in Brazil. J Comp Pathol. 2019;166:20–8. doi: 10.1016/j.jcpa.2018.10.171 30691602

[23] Biezus G, Grima de Cristo T, Bassi das Neves G, da Silva Casa M, Barros Brizola P, Silvestre Sombrio M, et al. Phylogenetic identification of feline leukemia virus A and B in cats with progressive infection developing into lymphoma and leukemia. Virus Res. 2023;329:199093. doi: 10.1016/j.virusres.2023.199093 36924831 PMC10194359

[24] Rojko JL, Hoover EA, Mathes LE, Olsen RG, Schaller JP. Pathogenesis of experimental feline leukemia virus infection. J Natl Cancer Inst. 1979;63(3):759–68. doi: 10.1093/jnci/63.3.759 224237

[25] Miyazawa T, Jarrett O. Feline leukaemia virus proviral DNA detected by polymerase chain reaction in antigenaemic but non-viraemic ('discordant’) cats. Arch Virol. 1997;142(2):323–32. doi: 10.1007/s007050050079 9125046

[26] Kaplan EL, Meier P. Nonparametric estimation from incomplete observations. J Am Stat Assoc. 1958;53(282):457–81. doi: 10.1080/01621459.1958.10501452

[27] Carvalho MS, Andreozzi VL, Codeço TC, Campos DP, Barbosa MTS, Shimakura SE. Análise de Sobreviência: teoria e aplicação em saúde. 2nd ed. Rio de Janeiro: Editora Fiocruz, 2011.

[28] Cox DR. Regression models and life-tables. J R Stat Soc Ser B. 1972;34(2):187–202. doi: 10.1111/j.2517-6161.1972.tb00899.x

[29] Cox DR, Oakes D. Analysis of Survival Data. London: Chapman & Hall. 1984.

[30] Weiss DJ, Wardrop KJ. Schalm’s veterinary hematology. 6th ed. Ames: Blackwell Publishing Ltd. 2010.

[31] Addie DD, Dennis JM, Toth S, Callanan JJ, Reid S, Jarrett O. Long-term impact on a closed household of pet cats of natural infection with feline coronavirus, feline leukaemia virus and feline immunodeficiency virus. Vet Rec. 2000;146(15):419–24. doi: 10.1136/vr.146.15.419 10811262

[32] Stützer B, Müller F, Majzoub M, Lutz H, Greene CE, Hermanns W, et al. Role of latent feline leukemia virus infection in nonregenerative cytopenias of cats. J Vet Intern Med. 2010;24(1):192–7. doi: 10.1111/j.1939-1676.2009.0417.x 19925574

[33] Stützer B, Simon K, Lutz H, Majzoub M, Hermanns W, Hirschberger J, et al. Incidence of persistent viraemia and latent feline leukaemia virus infection in cats with lymphoma. J Feline Med Surg. 2011;13(2):81–7. doi: 10.1016/j.jfms.2010.09.015 21131219 PMC10822315

[34] Spada E, Perego R, Sgamma EA, Proverbio D. Survival time and effect of selected predictor variables on survival in owned pet cats seropositive for feline immunodeficiency and leukemia virus attending a referral clinic in northern Italy. Prev Vet Med. 2018;150:38–46. doi: 10.1016/j.prevetmed.2017.12.001 29406082

[35] Beall MJ, Buch J, Clark G, Estrada M, Rakitin A, Hamman NT, et al. Feline Leukemia Virus p27 antigen concentration and proviral DNA load are associated with survival in naturally infected cats. Viruses. 2021;13(2):302. doi: 10.3390/v13020302 33671961 PMC7919025

[36] Hofmann-Lehmann R, Holznagel E, Ossent P, Lutz H. Parameters of disease progression in long-term experimental feline retrovirus (feline immunodeficiency virus and feline leukemia virus) infections: hematology, clinical chemistry, and lymphocyte subsets. Clin Diagn Lab Immunol. 1997;4(1):33–42. doi: 10.1128/cdli.4.1.33-42.1997 9008278 PMC170472

[37] Jackson ML, Haines DM, Meric SM, Misra V. Feline leukemia virus detection by immunohistochemistry and polymerase chain reaction in formalin-fixed, paraffin-embedded tumor tissue from cats with lymphosarcoma. Can J Vet Res. 1993;57(4):269–76. 8269365 PMC1263639

[38] Gabor LJ, Jackson ML, Trask B, Malik R, Canfield PJ. Feline leukaemia virus status of Australian cats with lymphosarcoma. Aust Vet J. 2001;79(7):476–81. doi: 10.1111/j.1751-0813.2001.tb13017.x 11549046

[39] Beatty JA, Tasker S, Jarrett O, Lam A, Gibson S, Noe-Nordberg A, et al. Markers of feline leukaemia virus infection or exposure in cats from a region of low seroprevalence. J Feline Med Surg. 2011;13(12):927–33. doi: 10.1016/j.jfms.2011.07.011 21880527 PMC10832971

[40] Ravi M, Wobeser GA, Taylor SM, Jackson ML. Naturally acquired feline immunodeficiency virus (FIV) infection in cats from western Canada: Prevalence, disease associations, and survival analysis. Can Vet J. 2010;51(3):271–6. 20514250 PMC2822370

[41] Hoover EA, Olsen RG, Hardy WD Jr, Schaller JP, Mathes LE. Feline leukemia virus infection: age-related variation in response of cats to experimental infection. J Natl Cancer Inst. 1976;57(2):365–9. doi: 10.1093/jnci/57.2.365 187771

[42] Leite-Filho RV, Panziera W, Bandinelli MB, Henker LC, da Conceição Monteiro K, Corbellini LG, et al. Epidemiological, pathological and immunohistochemical aspects of 125 cases of feline lymphoma in Southern Brazil. Vet Comp Oncol. 2020;18(2):224–30. doi: 10.1111/vco.12535 31461200

[43] Taylor SS, Goodfellow MR, Browne WJ, Walding B, Murphy S, Tzannes S, et al. Feline extranodal lymphoma: response to chemotherapy and survival in 110 cats. J Small Anim Pract. 2009;50(11):584–92. doi: 10.1111/j.1748-5827.2009.00813.x 19891724

[44] Marioni-Henry K, Van Winkle TJ, Smith SH, Vite CH. Tumors affecting the spinal cord of cats: 85 cases (1980-2005). J Am Vet Med Assoc. 2008;232(2):237–43. doi: 10.2460/javma.232.2.237 18275391

[45] Santagostino SF, Mortellaro CM, Boracchi P, Avallone G, Caniatti M, Forlani A, et al. Feline upper respiratory tract lymphoma: site, cyto-histology, phenotype, FeLV expression, and prognosis. Vet Pathol. 2015;52(2):250–9. doi: 10.1177/0300985814537529 24903757

[46] Meichner K, von Bomhard W. Patient characteristics, histopathological findings and outcome in 97 cats with extranodal subcutaneous lymphoma (2007-2011). Vet Comp Oncol. 2016;14 Suppl 1:8–20. doi: 10.1111/vco.12081 24410724

[47] Roccabianca P, Avallone G, Rodriguez A, Crippa L, Lepri E, Giudice C, et al. Cutaneous lymphoma at injection sites: pathological, immunophenotypical, and molecular characterization in 17 cats. Vet Pathol. 2016;53(4):823–32. doi: 10.1177/0300985815623620 26933095

[48] Shelton GH, Linenberger ML, Persik MT, Abkowitz JL. Prospective hematologic and clinicopathologic study of asymptomatic cats with naturally acquired feline immunodeficiency virus infection. J Vet Intern Med. 1995;9(3):133–40. doi: 10.1111/j.1939-1676.1995.tb03286.x 7674214 PMC7166774

[49] Abkowitz JL, Holly RD, Grant CK. Retrovirus-induced feline pure red cell aplasia. Hematopoietic progenitors are infected with feline leukemia virus and erythroid burst-forming cells are uniquely sensitive to heterologous complement. J Clin Invest. 1987;80(4):1056–63. doi: 10.1172/JCI113160 2821071 PMC442346

[50] Weiss DJ. Aplastic anemia in cats - clinicopathological features and associated disease conditions 1996-2004. J Feline Med Surg. 2006;8(3):203–6. doi: 10.1016/j.jfms.2005.11.002 16434225 PMC10832858

[51] Riedel N, Hoover EA, Gasper PW, Nicolson MO, Mullins JI. Molecular analysis and pathogenesis of the feline aplastic anemia retrovirus, feline leukemia virus C-Sarma. J Virol. 1986;60(1):242–50. doi: 10.1128/JVI.60.1.242-250.1986 3018287 PMC253922

[52] Hisasue M, Nishigaki K, Katae H, Yuri K, Mizuno T, Fujino Y, et al. Clonality analysis of various hematopoietic disorders in cats naturally infected with feline leukemia virus. J Vet Med Sci. 2000;62(10):1059–65. doi: 10.1292/jvms.62.1059 11073076

[53] Winzelberg Olson S, Hohenhaus AE. Feline non-regenerative anemia: diagnostic and treatment recommendations. J Feline Med Surg. 2019;21(7):615–31. doi: 10.1177/1098612X19856178 31234748 PMC10814193

[54] Hisasue M, Nagashima N, Nishigaki K, Fukuzawa I, Ura S, Katae H, et al. Myelodysplastic syndromes and acute myeloid leukemia in cats infected with feline leukemia virus clone33 containing a unique long terminal repeat. Int J Cancer. 2009;124(5):1133–41. doi: 10.1002/ijc.24050 19035458

[55] Shimoda T, Shiranaga N, Mashita T, Hasegawa A. A hematological study on thirteen cats with myelodysplastic syndrome. J Vet Med Sci. 2000;62(1):59–64. doi: 10.1292/jvms.62.59 10676891

[56] Hisasue M, Okayama H, Okayama T, Suzuki T, Mizuno T, Fujino Y, et al. Hematologic abnormalities and outcome of 16 cats with myelodysplastic syndromes. J Vet Intern Med. 2001;15(5):471–7. doi: 10.1111/j.1939-1676.2001.tb01577.x 11596735

[57] Kohn B, Weingart C, Eckmann V, Ottenjann M, Leibold W. Primary immune-mediated hemolytic anemia in 19 cats: diagnosis, therapy, and outcome (1998–2004). J Vet Int Med. 2006;20(1):159. doi: 10.1892/0891-6640(2006)20[159:pihaic]2.0.co;2 16496936

[58] Hofmann-Lehmann R, Cattori V, Tandon R, Boretti FS, Meli ML, Riond B, et al. Vaccination against the feline leukaemia virus: outcome and response categories and long-term follow-up. Vaccine. 2007;25(30):5531–9. doi: 10.1016/j.vaccine.2006.12.022 17240486

[59] Torres AN, Mathiason CK, Hoover EA. Re-examination of feline leukemia virus: host relationships using real-time PCR. Virology. 2005;332(1):272–83. doi: 10.1016/j.virol.2004.10.050 15661159

[60] Helfer-Hungerbuehler AK, Cattori V, Boretti FS, Ossent P, Grest P, Reinacher M, et al. Dominance of highly divergent feline leukemia virus A progeny variants in a cat with recurrent viremia and fatal lymphoma. Retrovirology. 2010;7:14. doi: 10.1186/1742-4690-7-14 20167134 PMC2837606

