# Supplementary material for: Survival analysis and clinical abnormalities in cats with progressive or regressive feline leukemia virus (FeLV) infection in Brazil
Source: PLoS One. 2025 Jul 1;20(7):e0322691. doi: 10.1371/journal.pone.0322691 (PMC12212530; doi:10.1371/journal.pone.0322691)
Supplement: S1 File — [Pages 1–4: Cox regression analysis for covariates associated with the survival curves of the cats belonging to the FeLV + P (n = 110) group]. [Variables in the equation: Age of cats at the time of inclusion in the study; Health status cats at the time of inclusion in the study; FIV co-infection]. (PDF) [file pone.0322691.s001.pdf]

```
COXREG Time
/STATUS=Outcome(1)
/CONTRAST (IVF)=Indicator(1) /CONTRAST
(healthstatus )=Indicator(1) /
METHOD=ENTER Age IVFhealthstatus
 /PLOT SURVIVAL
/PRINT=CI(95)
/CRITERIA=PIN(.05) POUT(.10) ITERATE(20).
```

Cox regression

Observations

Output created		19-MAY-2021 15:43:15
Comments		
Prohibited	Active dataset	DataSet1
	Filter	<none>
	Weighting	<none>
	Split File	<none>
	N of lines in job data file	110
Missing values Treatment	Definition of omission	Missing values user-defined are treated as missing.
Syntax		COXREG Time<br>/STATUS=Outcome(1)<br>/CONTRAST (IVF)<br>=Indicator(1)<br>/CONTRAST<br>(healthstatus)=Indicator<br>(1)<br>/METHOD=ENTER<br>Age FIV health status<br>/PLOT SURVIVAL<br>/PRINT=CI(95)<br>/CRITERIA=PIN(.05)<br>POUT(.10) ITERATE(20).
Resources	Processing time	00:00:00,50
	Elapsed time	00:00:00,47

Case Processing Summary

		N	Percentage
Avaliable cases under analysis	Event[a]	96	87.3%
	Censored	11	10.0%
	Total	107	97.3%
Dismissed cases	Cases with missing values	3	2.7%
	Negative time case	0	0.0%
	Cases censored before the first event	0	0.0%
	Total	3	2.7%
Overral		110	100.0%

a. Dependent Variable: Time

Categorical variable encodings[a,c]

		Frequency	(1)
health status[b]	, 00	10	0
	1.00	97	1
FIV[b]	, 00	99	0
	1.00	8	1

a. Categorical variable: health status (healthstatus)

b. Parameter coding

c. Categoricaç variable: FIV (FIV)

Block 0: Initial Block

Omnibus Test of Model Coefficient

-2 Log Likelihood
755,623

Block 1: Method = Enter

Omnibus Tests of Model Coefficients[a]

-2 Log Likelihood	Overall (score)			Change from previous step		
	Chi-square	df	Sig.	Chi-square	df	Sig.
744,677	9,063	3	, 028	10,946	3	, 012

Omnibus Tests of Model Coefficientsthe

Change from previous block		
**Chi-square**	df	Sig.
10,946	3	, 012

a. Beginning Block Number 1. Method = Enter

Variables in the equation

	B	SE	Wald	df	Sig.	Exp(B)	95.0% CI ..
							Lower
Age	- , 002	, 003	, 452	1	, 501	, 998	, 991
health status	1,127	, 399	7,976	1	, 005	3,087	1,412
FIV	, 221	, 374	, 351	1	, 554	1,248	, 600

Variables in the equation

	95.0% CI ...
	Upper
Age	1,004
health status	6,748
FIV	2,596

Covariate means

	Mean
Age	40,533
health status	, 907
FIV	, 075

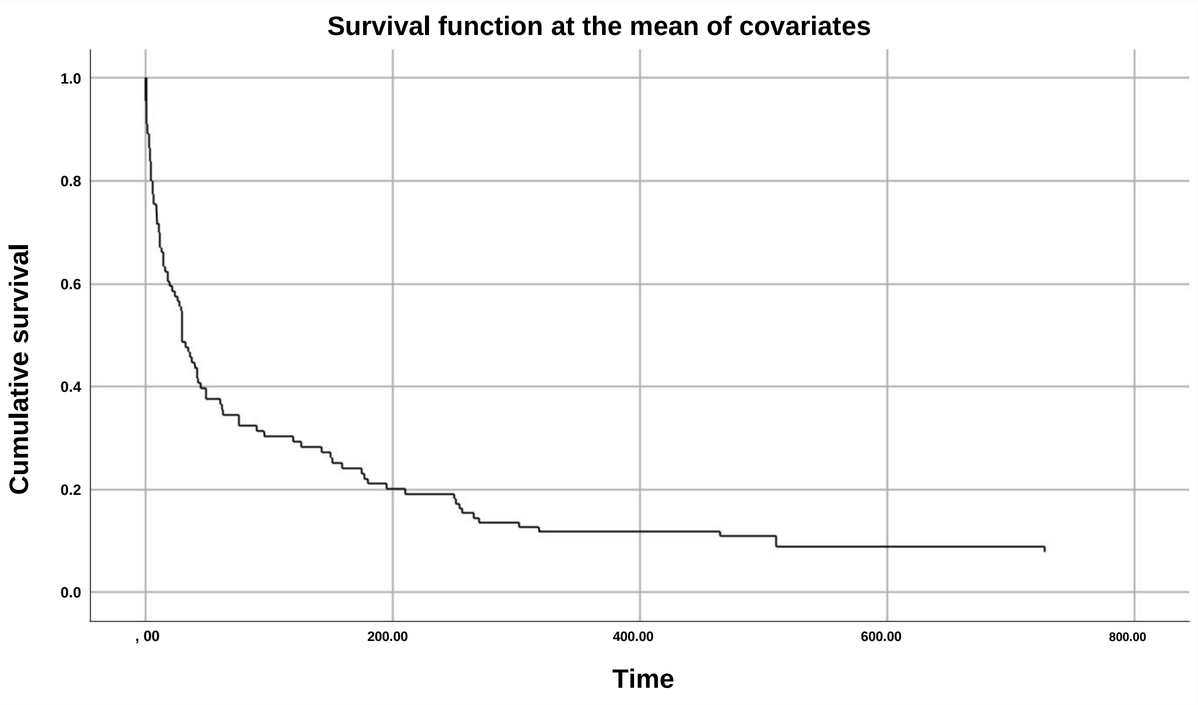
Survival function at the mean of covariates
Cumulative survival
1.0
0.8
0.6
0.4
0.2
0.0
, 00
200.00
400.00
600.00
800.00
Time
