# Supplementary material for: Survival analysis and clinical abnormalities in cats with progressive or regressive feline leukemia virus (FeLV) infection in Brazil
Source: PLoS One. 2025 Jul 1;20(7):e0322691. doi: 10.1371/journal.pone.0322691 (PMC12212530; doi:10.1371/journal.pone.0322691)

```
COXREG Time
/STATUS=Outcome(1)
/CONTRAST (healthstatus)=Indicator(1) /
CONTRAST (FIV)=Indicator(1)
/METHOD=ENTER Age FIV healthstatus /
PRINT=CI(95)
/CRITERIA=PIN(.05) POUT(.10) ITERATE(20).
```

Cox regression

Observations

Output created		19-MAY-2021 15:12:20
Comments		
Prohibited	Active dataset	DataSet2
	Filter	<none>
	Weighting	<none>
	Split File	<none>
	N of lines in job data file	23
Missing values treatment	Definition of omission	Missing values user-defined are treated as missing.
Syntax		COXREG Time /STATUS=Outcome(1) /CONTRAST (healthstatus)=Indicator (1) /CONTRAST (FIV) =Indicator(1) /METHOD=ENTER Age FIV health status /PRINT=CI(95) /CRITERIA=PIN(.05) POUT(.10) ITERATE(20).
Resources	Processing time	00:00:00,00
	Elapsed time	00:00:00,02

Case Processing Summary

		N	Percentage
Available cases under analysis	Event[a]	8	34.8%
	Censored	15	65.2%
	Total	23	100.0%
Dismissed cases	Cases with missing values	0	0.0%
	Negative time cases	0	0.0%
	Cases censored before the first event in a layer	0	0.0%
	Total	0	0.0%
Overall		23	100.0%

a. Dependent Variable: Time

Categorical variable encodings[a,c]

		Frequency	(1)
health status[b]	, 00	12	0
	1.00	11	1
FIV[b]	, 00	19	0
	1.00	4	1

a. Categorical variable: health status (healthstatus)

b. Parameter coding

c. Categorical variable: FIV (FIV)

Block 0: Initial Block

Omnibus Test of Model Coefficient

-2 Log Likelihood
47,143

Block 1: Method = Enter

Omnibus Tests of Model Coefficients[a]

-2 Log Likelihood	Overall (score)			Change from previous step		
	Chi-square	df	Sig.	Chi-square	df	Sig.
35,413	13,136	3	, 004	11,731	3	, 008

Omnibus Tests of Model Coefficients[a]

Change from previous block		
Chi-square	df	**Sig.**
11,731	3	**,008**

a. Beginning Block Number 1. Method = Enter

Variables in the equation

	B	SE	Wald	df	Sig.	Exp(B)	95.0% CI .. Lower
Age	, 005	, 005	, 825	1	, 364	1,005	, 994
health status	2,590	1,138	5,182	1	, 023	13,333	1,433
FIV	- 1,133	1,082	1,097	1	, 295	, 322	, 039

Variables in the equation

	95.0% CI ... Upper
Age	1,015
health status	124,027
FIV	2,684

Covariate means

	Means
Age	43,652
health status	, 478
FIV	, 174

```
NEW FILE.
DATASET NAMEDataset3 WINDOW=FRONT.
COXREG Time
  /STATUS=Outcome(1)
  /CONTRAST (healthstatus )=Indicator(1) /
  METHOD=ENTER Agehealthstatus /
  PRINT=CI(95)
  /CRITERIA=PIN(.05) POUT(.10) ITERATE(20).
```

Cox regression

Observations

Output created		19-MAY-2021 15:15:02
Comments		
Prohibited	Active dataset	DataSet3
	Filter	<none>
	Weighting	<none>
	Split File	<none>
	N of lines in job data file	21
Missing values treatment	Definition of omission	Missing values user-defined are treated as missing.
Syntax		COXREG Team /STATUS=Outcome(1) /CONTRST (healthstatus)=Indicator (1) /METHOD=ENTER Age health status /PRINT=CI(95) /CRITERIA=PIN(.05) POUT(.10) ITERATE(20).
Resources	Processing time	00:00:00,00
	Elapsed time	00:00:00,01

[Dataset3]

Case Processing Summary

		N	Percentage
Cases available under analysis	Event[a]	6	28.6%
	Censored	15	71.4%
	Total	21	100.0%
Dismissed cases	Cases with missing values	0	0.0%
	Negative time cases	0	0.0%
	Cases censored before the first event	0	0.0%
	Total	0	0.0%
Overral		21	100.0%

a. Dependent Variable: Time

Categorical variable encodings[a]

		Frequency	(1)
health status[b]	, 00	8	0
	1.00	13	1

a. Categorical variable: health status (healthstatus)

b. Parameter coding

Block 0: Initial Block

Tests of Omnibus Model Coefficient

-2 Log Likelihood
34,833

Block 1: Method = Enter

Omnibus Tests of Model Coefficients[a]

-2 Log Likelihood	Overall (score)			Change from previous step		
	Chi-square	df	Sig.	Chi-square	df	Sig.
32,045	2,712	2	, 258	2,787	2	, 248

Omnibus Tests of Model Coefficients[a]

Change from previous block		
Chi-square	df	Sig.
2,787	2	, 248

. Beginning Block Number 1. Method = Ente

Variables in the equation

	B	SE	Wald	df	Sig.	Exp(B)	95.0% CI ..
							Lower
Age	, 008	, 009	, 723	1	, 395	1,008	, 990
health status	1,013	1,192	, 722	1	, 395	2,754	, 266

Variables in the equation

	95.0% CI ...
	Upper
Age	1,025
health status	28,483

Covariate means

	Means
Age	68,190
health status	, 619

```
DATASET ACTIVATE Dataset1 .
COXREG Time
/STATUS=Outcome(1)
/METHOD=ENTER Age
/PLOT SURVIVAL
/PRINT=CI(95)
/CRITERIA=PIN(.05) POUT(.10) ITERATE(20).
```

Cox regression

Observations

Output created		19-MAY-2021 15:21:16
Comments		
Prohibited	Active dataset	DataSet1
	Filter	<none>
	Weighting	<none>
	Split File	<none>
	N of lines in job data file	110
Missing values treatment	Definition of omission	Missing values user-defined are treated as missing.
Syntax		COXREG Time /STATUS=Outcome(1) /METHOD=ENTER Age /PLOT SURVIVAL /PRINT=CI(95) /CRITERIA=PIN(.05) ...
Resources	Processing time	00:00:00,50
	Elapsed time	00:00:00,37

[Dataset1]

Case Processing Summary

		N	Percentage
Cases available under analysis	Event[a]	96	87.3%
	Censored	11	10.0%
	Total	107	97.3%
Dismissed cases	Cases with missing values	3	2.7%
	Negative cases time	0	0.0%
	Cases censored before the first event	0	0.0%
	Total	3	2.7%
Overral		110	100.0%

a. Dependent Variable: Time

Block 0: Initial Block

Omnibus Test of Model Coefficient

-2 Log Likelihood
755,623

Block 1: Method = Enter

Omnibus Tests of Model Coefficients[a]

-2Log Likelihood	Overall (score)			Change from previous step		
	Chi-square	df	Sig.	Chi-square	df	Sig.
755,442	, 178	1	, 673	, 182	1	, 670

Omnibus Tests of Model Coefficientsthe

Change from previous block		
Chi-square	df	Sig.
, 182	1	, 670

a. Begininh Block Number 1. Method = Enter

Variables in the equation

	B	SE	Wald	df	Sig.	Exp(B)	95.0% CI for Exp(B) Lower	Upper
Age	- , 001	, 003	, 178	1	, 673	, 999	, 992	1,005

Covariate Means

	Mean
Age	40,533

Survival function at the mean of covariates

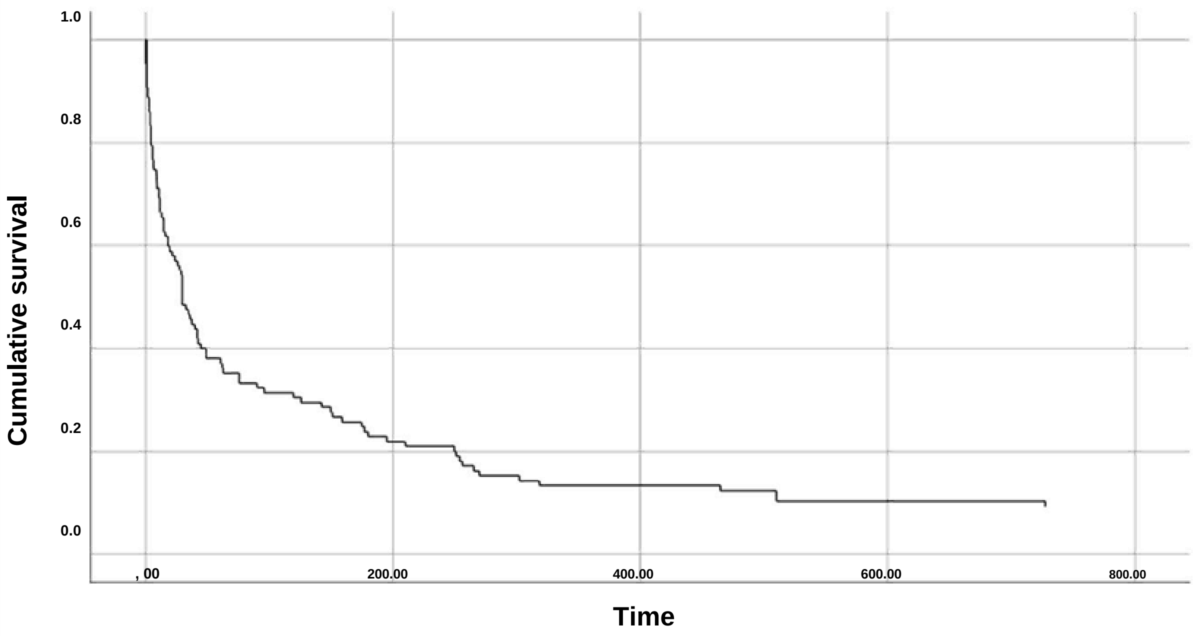

Supplement: S2 File — [Pages 1–3: Cox regression analysis for covariates associated with the survival curves of the cats belonging to the FeLV + R (n = 23)], [Pages 4–8: Cox regression analysis for covariates associated with the survival curves of the cats belonging to the control (n = 21) group]. [Variables in the equation: Age of cats at the time of inclusion in the study; Health status cats at the time of inclusion in the study; FIV co-infection]. (PDF) [file pone.0322691.s002.pdf]
