# Supplementary material for: Survival analysis and clinical abnormalities in cats with progressive or regressive feline leukemia virus (FeLV) infection in Brazil
Source: PLoS One. 2025 Jul 1;20(7):e0322691. doi: 10.1371/journal.pone.0322691 (PMC12212530; doi:10.1371/journal.pone.0322691)
Supplement: S3 File — [Pages 1–12: Kaplan–Meier analysis and survival curves obtained for cats with progressive FeLV infection (FeLV + P; n = 110), cats with regressive infection (FeLV + R; n = 23), and the control group (n = 21)]. [Pages 13–17: Cox regression analysis for covariates associated with the survival curves of the total cats]. [Variables in the equation: Age of cats at the time of inclusion in the study; Health status cats at the time of inclusion in the study; FIV co-infection; Groups in the study (FeLV + P, FeLV + R and Control)]. (PDF) [file pone.0322691.s003.pdf]

Your temporary usage period for IBMSPSS Statistics will expire in 5344 days.

```
NEW FILE.
DATASET NAMEDataSet1 WINDOW=FRONT. KM Time BY
Treatment
  /STATUS=Outcome(1)
  /PRINT TABLE MEAN
  /PLOT SURVIVAL
  /TEST LOGRANK BRESLOW TARONE /
  COMPARE OVERALL POOLED.
```

Kaplan-Meier

Observations

Output created		14-MAY-2021 17:43:34
Comments		
Prohibited	Active dataset	DataSet1
	Filter	<none>
	Weighting	<none>
	Split File	<none>
	N of lines in job data file	154
Missing value treatment	Definition of omission	Missing values user-defined are treated as missing.
	Cases used	The statistics are based on all cases with valid data for all analysis variables.
Syntax		KM Time BY Treatment /STATUS=Outcome(1) /PRINT TABLE MEAN /PLOT SURVIVAL /TEST LOGRANK BRESLOW TARONE /COMPARE OVERALL POOLED.
Resources	Processing time	00:00:04.06
	Elapsed time	00:00:02.72

[Dataset1]

Case Processing Summary

Treatment	N total	Selected Cases	Missing Cases N	Missing Cases Percent
1.00	109	98	11	10.1%
2.00	23	8	15	65.2%
3.00	21	6	15	71.4%
Total	153	112	41	26.8%

Survival table

Treatment		Time	Status	Cumulative proportion surviving time Estimate	Cumulative proportion surviving time Error Error	N of cumulative events
1.00	1	. 000	1.00	.	.	1
	2	. 000	1.00	.	.	2
	3	. 000	1.00	.	.	3
	4	. 000	1.00	.	.	4
	5	. 000	1.00	. 954	. 020	5
	6	1.000	1.00	.	.	6
	7	1.000	1.00	.	.	7
	8	1.000	1.00	.	.	8
	9	1.000	1.00	.	.	9
	10	1.000	1.00	. 908	. 028	10
	11	2.000	1.00	.	.	11
	12	2.000	1.00	. 890	. 030	12
	13	3.000	1.00	.	.	13
	14	3.000	1.00	.	.	14
	15	3.000	1.00	. 862	. 033	15
	16	4.000	1.00	.	.	16
	17	4.000	1.00	.	.	17
	18	4.000	1.00	. 835	. 036	18
	19	5.000	1.00	.	.	19
	20	5.000	1.00	.	.	20
	21	5.000	1.00	.	.	21
	22	5.000	1.00	. 798	. 038	22
	23	6.000	1.00	.	.	23
	24	6.000	1.00	.	.	24
	25	6.000	1.00	. 771	. 040	25
	26	7.000	1.00	.	.	26
	27	7.000	1.00	. 752	. 041	27
	28	9.000	1.00	.	.	28
	29	9.000	1.00	.	.	29

Survival table

Treatment		Remaining cases
1.00	1	108
	2	107
	3	106
	4	105
	5	104
	6	103
	7	102
	8	101
	9	100
	10	99
	11	98
	12	97
	13	96
	14	95
	15	94
	16	93
	17	92
	18	91
	19	90
	20	89
	21	88
	22	87
	23	86
	24	85
	25	84
	26	83
	27	82
	28	81
	29	80

Survival table

Treatment	Time	Status	Comulative proportion surviving time Estimate	Error Error	N of cumulative events
30	9.000	1.00	.	.	30
31	9.000	1.00	.	.	31
32	9.000	1.00	. 706	. 044	32
33	11.000	1.00	.	.	33
34	11.000	1.00	. 688	. 044	34
35	12.000	1.00	.	.	35
36	12.000	1.00	.	.	36
37	12.000	1.00	. 661	. 045	37
38	13.000	1.00	. 651	. 046	38
39	15.000	1.00	.	.	39
40	15.000	1.00	.	.	40
41	15.000	1.00	. 624	. 046	41
42	16.000	1.00	. 615	. 047	42
43	18.000	1.00	.	.	43
44	18.000	1.00	. 596	. 047	44
45	20.000	1.00	. 587	. 047	45
46	22.000	1.00	. 578	. 047	46
47	24.000	1.00	. 569	. 047	47
48	26.000	1.00	. 560	. 048	48
49	28.000	1.00	. 550	. 048	49
50	28.000	. 00	.	.	49
51	29.000	1.00	. 541	. 048	50
52	30.000	1.00	.	.	51
53	30.000	1.00	.	.	52
54	30.000	1.00	.	.	53
55	30.000	1.00	.	.	54
56	30.000	1.00	.	.	55
57	30.000	1.00	. 485	. 048	56
58	33.000	1.00	. 476	. 048	57
59	35.000	1.00	. 466	. 048	58
60	36.000	1.00	. 457	. 048	59
61	38.000	1.00	. 448	. 048	60
62	40.000	1.00	. 439	. 048	61
63	42.000	1.00	.	.	62
64	42.000	1.00	. 420	. 047	63
65	43.000	1.00	. 411	. 047	64
66	45.000	1.00	. 401	. 047	65

Survival table

Treatment	Remaining cases
30	79
31	78
32	77
33	76
34	75
35	74
36	73
37	72
38	71
39	70
40	69
41	68
42	67
43	66
44	65
45	64
46	63
47	62
48	61
49	60
50	59
51	58
52	57
53	56
54	55
55	54
56	53
57	52
58	51
59	50
60	49
61	48
62	47
63	46
64	45
65	44
66	43

Survivel table

Treatment	Time	Status	Comulative proportion surviving time Estimate	Error Error	N of cumulative events
67	49.000	1.00	.	.	66
68	49.000	1.00	. 383	. 047	67
69	61.000	1.00	. 373	. 047	68
70	62.000	1.00	. 364	. 046	69
71	63.000	1.00	. 355	. 046	70
72	76.000	1.00	.	.	71
73	76.000	1.00	. 336	. 045	72
74	90.000	1.00	. 327	. 045	73
75	96.000	1.00	. 317	. 045	74
76	120.000	1.00	. 308	. 044	75
77	126.000	1.00	. 299	. 044	76
78	143.000	1.00	. 289	. 044	77
79	145.000	1.00	. 280	. 043	78
80	150.000	1.00	. 271	. 043	79
81	151.000	1.00	. 261	. 042	80
82	159.000	1.00	. 252	. 042	81
83	175.000	1.00	. 243	. 041	82
84	177.000	1.00	. 233	. 041	83
85	180.000	1.00	. 224	. 040	84
86	195.000	1.00	. 215	. 040	85
87	210.000	1.00	. 205	. 039	86
88	250.000	1.00	. 196	. 038	87
89	251.000	1.00	. 187	. 038	88
90	254.000	1.00	. 177	. 037	89
91	256.000	1.00	. 168	. 036	90
92	266.000	1.00	. 159	. 035	91
93	270.000	1.00	. 149	. 034	92
94	302.000	1.00	. 140	. 033	93
95	318.000	1.00	. 131	. 033	94
96	465.000	1.00	. 121	. 032	95
97	480.000	. 00	.	.	95
98	510.000	1.00	.	.	96
99	510.000	1.00	. 101	. 029	97
100	727.000	1.00	. 091	. 028	98
101	730.000	. 00	.	.	98
102	730.000	. 00	.	.	98
103	730.000	. 00	.	.	98

Survival table

Treatment	Remaining cases
67	42
68	41
69	40
70	39
71	38
72	37
73	36
74	35
75	34
76	33
77	32
78	31
79	30
80	29
81	28
82	27
83	26
84	25
85	24
86	23
87	22
88	21
89	20
90	19
91	18
92	17
93	16
94	15
95	14
96	13
97	12
98	11
99	10
100	9
101	8
102	7
103	6

Survivel table

Treatment		Time	Status	Cumulative proportion surviving time Estimate	Error Error	N of cumulative events
	104	730.000	. 00	.	.	98
	105	730.000	. 00	.	.	98
	106	730.000	. 00	.	.	98
	107	730.000	. 00	.	.	98
	108	730.000	. 00	.	.	98
	109	730.000	. 00	.	.	98
2.00	1	. 000	1.00	. 957	. 043	1
	2	1.000	1.00	.	.	2
	3	1.000	1.00	. 870	. 070	3
	4	3.000	1.00	. 826	. 079	4
	5	16.000	1.00	. 783	. 086	5
	6	30.000	. 00	.	.	5
	7	48.000	1.00	. 737	. 092	6
	8	50.000	1.00	. 691	. 097	7
	9	365.000	1.00	. 645	. 101	8
	10	730.000	. 00	.	.	8
	11	730.000	. 00	.	.	8
	12	730.000	. 00	.	.	8
	13	730.000	. 00	.	.	8
	14	730.000	. 00	.	.	8
	15	730.000	. 00	.	.	8
	16	730.000	. 00	.	.	8
	17	730.000	. 00	.	.	8
	18	730.000	. 00	.	.	8
	19	730.000	. 00	.	.	8
	20	730.000	. 00	.	.	8
	21	730.000	. 00	.	.	8
	22	730.000	. 00	.	.	8
	23	730.000	. 00	.	.	8
3.00	1	5.000	1.00	. 952	. 046	1
	2	8.000	1.00	. 905	. 064	2
	3	12.000	1.00	. 857	. 076	3
	4	180.000	1.00	. 810	. 086	4
	5	305.000	1.00	. 762	. 093	5
	6	425.000	. 00	.	.	5
	7	455.000	1.00	. 711	. 100	6
	8	455.000	. 00	.	.	6

Survival table

Treatment		Remaining cases
	104	5
	105	4
	106	3
	107	2
	108	1
	109	0
2.00	1	22
	2	21
	3	20
	4	19
	5	18
	6	17
	7	16
	8	15
	9	14
	10	13
	11	12
	12	11
	13	10
	14	9
	15	8
	16	7
	17	6
	18	5
	19	4
	20	3
	21	2
	22	1
	23	0
3.00	1	20
	2	19
	3	18
	4	17
	5	16
	6	15
	7	14
	8	13

Survival table

Treatment	Time	Status	Cumulative proportion surviving time		N of cumulative events
			Estimate	Error Error	
9	665.000	. 00	.	.	6
10	730.000	. 00	.	.	6
11	730.000	. 00	.	.	6
12	730.000	. 00	.	.	6
13	730.000	. 00	.	.	6
14	730.000	. 00	.	.	6
15	730.000	. 00	.	.	6
16	730.000	. 00	.	.	6
17	730.000	. 00	.	.	6
18	730.000	. 00	.	.	6
19	730.000	. 00	.	.	6
20	730.000	. 00	.	.	6
21	730.000	. 00	.	.	6

Survival table

Treatment	N of remaining cases
9	12
10	11
11	10
12	9
13	8
14	7
15	6
16	5
17	4
18	3
19	2
20	1
21	0

Means and Medians for Survival Time

Treatment	Mean[a]				Median	
			95% Confidence interval			
	Estimate	Error Error	Lower bound	Upper bound	Estimate	Error Error
1.00	141.497	21.611	99.140	183.855	30.000	4.674
2.00	492.714	68.984	357.505	627.922	.	.
3.00	566.508	59.665	449.565	683.451	.	.
Overall	252.289	24.813	203.656	300.923	50.000	22.018

Means and Medians for Survival Time

Treatment	Median	
	95% Confidence interval	
	Lower bound	Upper bound
1.00	20.840	39.160
2.00	.	.
3.00	.	.
Overall	6.844	93.156

a. Estimation is limited to the longest survival time if it is censored.

Overall comparisons

	Chi-square	df	Sig.
Log Rank (Mantel-Cox)	42.114	2	. 000
Breslow (Generalized Wilcoxon)	28.317	2	. 000
Tarone-Ware	35.337	2	. 000

Test of equality of survival distributions for the different treatment levels.

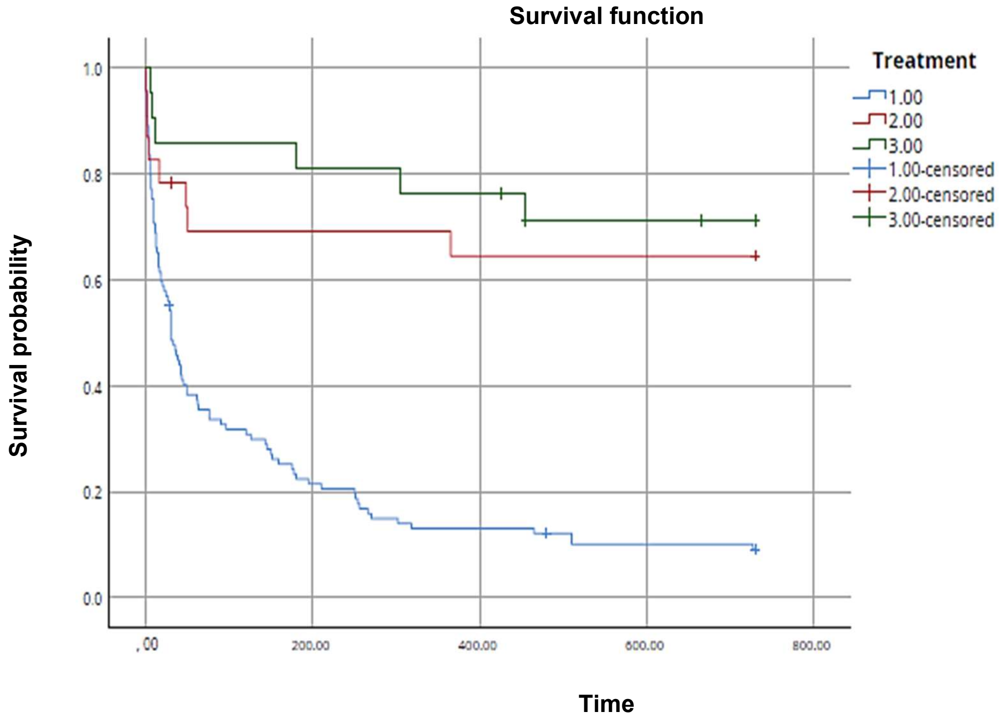


```
COXREG Time
/STATUS=Outcome(1)
/PATTERN BY Treatment
/CONTRAST (IVF)=Indicator(1)
/CONTRAST (healthstatus)=Indicator(1)
/CONTRAST (Treatment)=Indicator
/METHOD=ENTER Age healthstatus Treatment FIV
/PLOT SURVIVAL
/PRINT=CI(95)
/CRITERIA=PIN(.05) POUT(.10) ITERATE(20).
```

Cox regression

Observations

Output created		14-MAY-2021 17:47:04
Comments		
Prohibited	Active dataset	DataSet1
	Filter	<none>
	Weighting	<none>
	Split File	<none>
	N of lines in job data file	154
Missing values treatment	Definition of omission	Missing values user-defined are treated as missing.
Syntax		COXREG Time<br>/STATUS=Outcome(1)<br>/PATTERN BY Treatment<br>/CONTRAST (IVF) =Indicator(1)<br>/CONTRAST (healthstatus)=Indicator (1)<br>/CONTRAST (Treatment)=Indicator<br>/METHOD=ENTER Age health status FIV Treatment<br>/PLOT SURVIVAL<br>/PRINT=CI(95)<br>/CRITERIA=PIN(.05) POUT(.10) ITERATE(20).
Resources	Processing time	00:00:01,06
	Elapsed time	00:00:00,54

Case Processing Summary

		N	Percentage
Available cases under analysis	Event[a]	110	71.4%
	Censored	41	26.6%
	Total	151	98.1%
Dismissed cases	Cases with missing values	3	1.9%
	Negative time cases	0	0.0%
	Cases censored before the first event	0	0.0%
	Total	3	1.9%
Overall		154	100.0%

a. Dependent Variable: Time

Categorical variable encodings[a,c,d]

		Frequency	(1)	(2)
health status[b]	. 00	30	0	
	1.00	121	1	
Treatment[b]	1.00	107	1	0
	2.00	23	0	1
	3.00	21	0	0
FIV[b]	. 00	137	0	
	1.00	14	1	

a. Categorical variable: health status (healthstatus)

b. Parameter coding

c. Categorical variable: Treatment (Treatment)

d. Categorical variable: FIV (FIV)

Block 0: Beginning Block

Omnibus Test of Model Coefficient

-2 Log Likelihood
989.806

Block 1: Method = Enter

Omnibus Tests of Model Coefficients[a]

-2 Log Likelihood	Overall (score)			Change from previous step		
	Chi-square	df	Sig.	Chi-square	df	Sig.
921.317	55.409	5	. 000	68.490	5	. 000

Omnibus Tests of Model Coefficients[a]

Change from previous block		
Chi-square	df	Sig.
68.490	5	. 000

a. Beginning Block Number 1. Method = Enter

Variables in the equation

	B	SE	Wald	df	Sig.	Exp(B)	95.0% CI ..
							Lower
Age	. 001	. 003	. 294	1	. 588	1.001	. 996
health status	1.398	. 372	14.091	1	. 000	4.046	1.950
Treatment			18.586	2	. 000		
Treatment(1)	1.699	. 438	15.036	1	. 000	5.469	2.317
Treatment(2)	. 782	. 555	1.984	1	. 159	2.186	. 736
FIV	. 042	. 339	. 015	1	. 901	1.043	. 536

Variables in the equation

	95.0% CI ...
	Upper
Age	1.007
health status	8.393
Treatment	
Treatment(1)	12.908
Treatment(2)	6.494
FIV	2.029

Covariate means and pattern values

		Standard		
	Mean	1	2	3
Age	44.854	44.854	44.854	44.854
health status	. 801	. 801	. 801	. 801
Treatment(1)	. 709	1.000	. 000	. 000
Treatment(2)	. 152	. 000	1.000	. 000
FIV	. 093	. 093	. 093	. 093

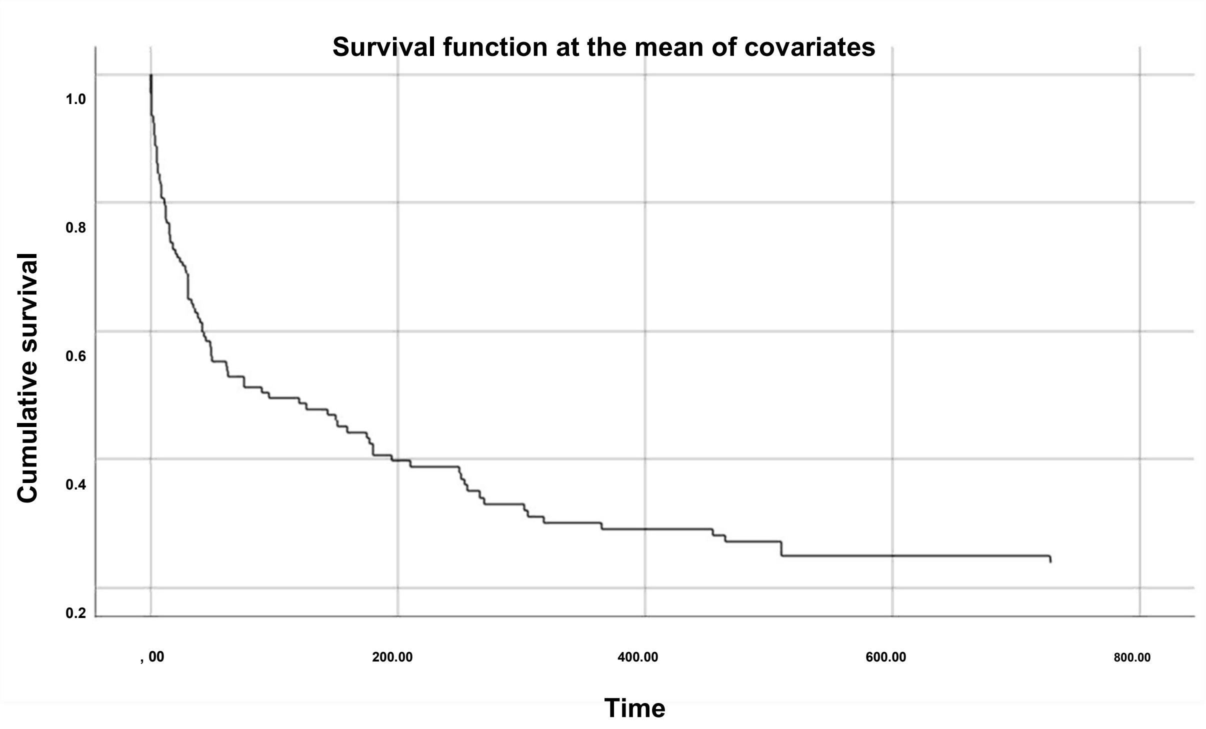

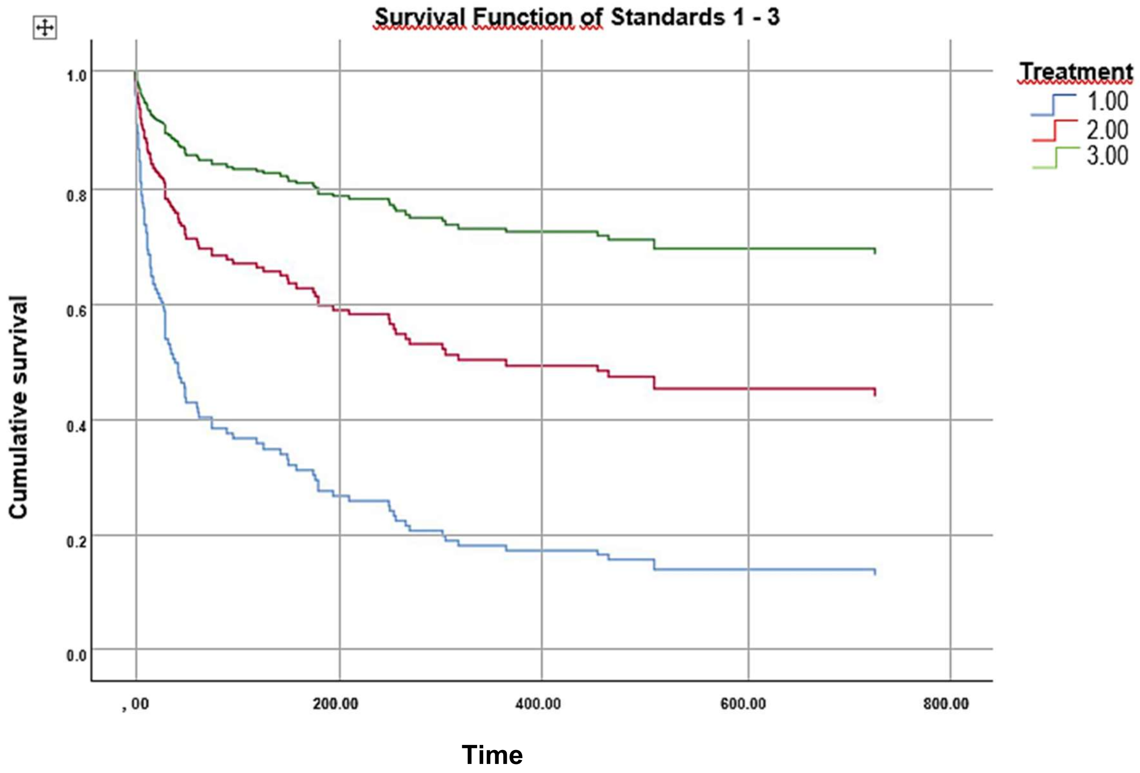
Survival Function of Standards 1 - 3
Treatment
1.00
2.00
3.00
Cumulative survival
1.0
0.8
0.6
0.4
0.2
0.0
, 00
200.00
400.00
600.00
800.00
Time
